# Periodontal reconstruction by heparan sulfate mimetic-based matrix therapy in *Porphyromonas gingivalis*-infected mice

**DOI:** 10.1016/j.heliyon.2018.e00719

**Published:** 2018-08-06

**Authors:** Benjamin R. Coyac, Laurent Detzen, Philippe Doucet, Brigitte Baroukh, Annie Llorens, Martine Bonnaure-Mallet, Marjolaine Gosset, Denis Barritault, Marie-Laure Colombier, Jean-Louis Saffar

**Affiliations:** a EA2496 Laboratoire Pathologies, Imagerie et Biothérapies oro-faciales, Faculté de Chirurgie Dentaire, Université Paris Descartes, Sorbonne Paris Cité, Montrouge, France; b Assistance Publique – Hôpitaux de Paris, Paris, France; c Private Practice in Periodontics, Paris, France; d U-1241 Inserm – U-1341 Inra, Université de Rennes 1, Rennes, France; e OTR3, Paris, France

**Keywords:** Dentistry, Bioengineering, Biotechnology, Cell biology, Immunology, Physiology

## Abstract

**Background:**

Periodontitis is a set of chronic inflammatory diseases affecting the supporting structures of the teeth, during which a persistent release of lytic enzymes and inflammatory mediators causes a self-perpetuating vicious cycle of tissue destruction and repair. A matrix-based therapy using a heparan sulfate (HS) analogue called ReGeneraTing Agent (RGTA) replaces destroyed HS by binding to available heparin-binding sites of structural molecules, leading to restoration of tissue homeostasis in several inflammatory tissue injuries, including a hamster periodontitis model.

**Methods:**

The ability of RGTA to restore the periodontium was tested in a model of *Porphyromonas gingivalis*-infected Balb/cByJ mice. After 12 weeks of disease induction, mice were treated weekly with saline or RGTA (1.5 mg/kg) for 8 weeks. Data were analyzed by histomorphometry.

**Results:**

RGTA treatment restored macroscopic bone loss. This was related to (1) a significant reduction in gingival inflammation assessed by a decrease in infiltrated connective tissue, particularly in cells expressing interleukin 1ß, an inflammatory mediator selected as a marker of inflammation; (2) a normalization of bone resorption parameters, i.e. number, activation and activity of osteoclasts, and number of preosteoclasts; (3) a powerful bone formation reaction. The Sharpey's fibers of the periodontal ligament recovered their alkaline phosphatase coating. This was obtained while *P. gingivalis* infection was maintained throughout the treatment period.

**Conclusions:**

RGTA treatment was able to control the chronic inflammation characteristic of periodontitis and blocked destruction of periodontal structures. It ensured tissue regeneration with recovery of the periodontium's anatomy.

## 1 Introduction

Periodontitis is a set of chronic inflammatory diseases characterized by gingival inflammation, pocket formation, alveolar bone loss, and attachment apparatus destruction [1]. Bacteria and their products trigger the release of host proteolytic enzymes and cytokines that mediate destruction of periodontal structures [2].

During inflammation, the organism responds to harmful stimuli, initiating a cascade of events which lead to cell death and matrix degradation, followed by cell proliferation, matrix repair and tissue remodeling [3, 4]. During the destructive phase, resident and inflammatory cells release proteases and glycanases that mediate degradation of collagens and glycosaminoglycans (GAGs) of the extracellular matrix (ECM), jeopardizing cell-ECM and cell-cell communication [3]. The inflammatory reaction also supplies signaling molecules such as growth factors, cytokines, and chemokines which stimulate its resolution and enable tissue repair [4]. The repair process that is initiated results in a reconstructed ECM that does not completely regenerate the injured tissue, but instead leads to a scar. In chronic inflammation, the progress to resolution is hindered. Local stimuli, such as pocket bacteria in the case of periodontitis, and products of matrix degradation prolong the inflammatory reaction, causing a persistent release of lytic enzymes and inflammatory mediators [3, 4, 5, 6, 7]. This exacerbates ECM destruction and causes sustained degradation of structure and repair-promoting molecules. Consequently, a self-perpetuating vicious cycle of cell/ECM destruction and repair settles in [3, 4].

Among ECM structure molecules, heparan sulfates (HS) regulate tissue homeostasis by mediating and integrating cell-cell and cell-matrix communication [8, 9, 10]. Indeed, a great number of signaling molecules or communication factors bind to HS. HS store, protect, and regulate the bioavailability of communication factors intervening in homeostatic cell replacement or tissue repair [11]. During inflammation, HS degradation releases cytokines that aggravate tissue injury. The concept of replacing degraded natural HS of injured matrix to restore the extracellular microenvironment and tissue homeostasis led to the engineering of polysaccharides mimicking HS, named RGTA (ReGeneraTing Agents). By binding to ECM structure and signaling proteins, RGTA prevents their proteolysis, favors their neo-synthesis, and enhances the speed and quality of tissue repair [9, 10].

In a hamster model of periodontitis, RGTA treatment acted into two steps: it first reduced gingival inflammation, restructured gingival tissues, and controlled pathologic bone remodeling by decreasing excessive osteoclastic resorption and enhancing osteogenic cell recruitment. In a second step, RGTA treatment rebuilt lost alveolar bone, increased periodontal ligament (PDL) cellularity and cementum thickness, and regenerated a functional attachment apparatus with Sharpey's fibers inserted in acellular cementum [12, 13, 14]. Notably, these changes occurred without any attempt to control the bacterial compoentn.

In this study, using a model of *Porphyromonas gingivalis (P. gingivalis)*-infected mice, our aim was to test the effectiveness of RGTA treatment on (1) the bone metabolism, i.e. bone resorption and formation, with an emphasis on the recruitment and activity of the osteoclastic lineage, and (2) the inflammatory response, by evaluating the recruitment of cells expressing interleukin 1ß (IL-1ß) which we used as marker of inflammation, as it is the main cytokine expressed in the gingival tissues during periodontitis [15].

## 2 Materials & methods

### 2.1 Animals and experimental protocol design

This study was approved by the Animal Experimentation Ethics committee of Université Paris Descartes (approval number CEEA34.MG.036.12) and complied with European Union recommendations on laboratory animal care (EU directive 2010/63/EU).

The animals were maintained in a temperature-controlled (25 °C) facility with a 12 h light/dark cycle. Prior to killing, animals were anesthetized with xylazine (100 mg/kg b.w., Centravet, Maisons Alfort, France) and ketamine (80 mg/kg b.w., Centravet, Maisons Alfort, France).

**Fig. 1:**
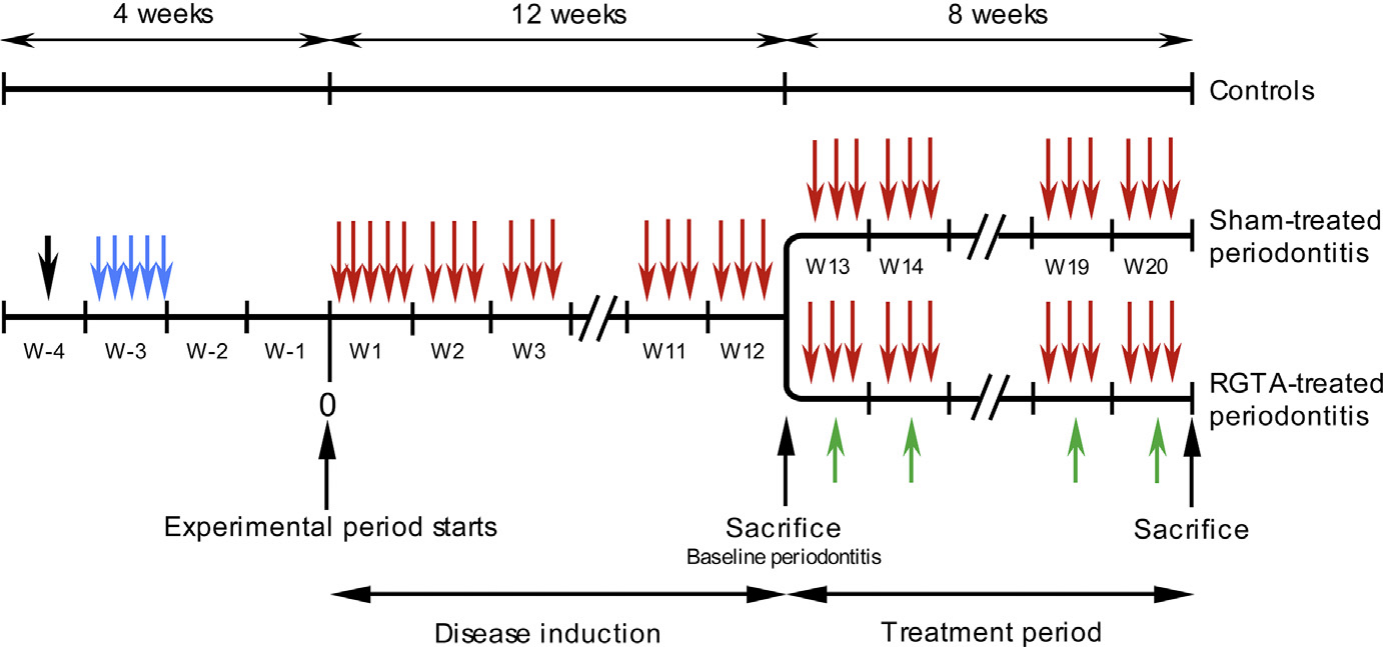
Timeline of the experimental protocol. After oral flora depression (black arrow) the mice were orally infected daily for 5 days with *A. viscosus*, strain CIP 103147 (blue arrows). Two weeks later (start of the experimental period), *P. gingivalis* was orally inoculated for five days during the first week (red arrows). For the following 11 weeks, *P. gingivalis* (strain W83) was inoculated 3 times per week (disease induction phase). At the end of the 12^th^ week, control (n = 15) and *P gingivalis*-inoculated mice (n = 15) were killed to evaluate the macroscopic alveolar bone status. The remaining periodontitis-affected mice were separated into 2 groups (treatment phase). One group (n = 15) was inoculated 3 times per week and received weakly saline intramuscular injections for 8 weeks (sham-treated periodontitis group). The other group (n = 30) was inoculated 3 times per week and received weekly intramuscular injections of RGTA in saline (1.5 mg/kg bw) for 8 weeks (RGTA-treated group (green arrows). At the end of the treatment period, the mice from the control (n = 15), sham-treated (n = 15) and RGTA-treated (n = 30) groups were killed.

Ninety 10-week-old male Balb/cByJ (Jackson strain, Charles River Laboratories, L'Arbresle, France) mice (*mus musculus*) were used. They were separated into 2 lots. One lot (30 mice) was used as a control and fed a standard diet in pellets (M20, Dietex, Argenteuil, France) for the entire course of the study. The other lot (60 mice) was given the following treatment: 4 days of the same standard diet pulverized and mixed with streptomycine sulfate (30 mg/kg b.w., Sigma-Aldrich Corp, Lyon, France) to depress the oral flora. Two days later, the diet was switched to a high carbohydrate diet composed of finely ground standard M20 diet (60 %) and glucose (40%, Sigma-Aldrich Corp, Lyon, France). *Actinomyces viscosus* (*A. viscosus*, strain CIP 103147, Institut Pasteur, Paris, France) suspended in saline was inoculated by oral gavage to the animals for 5 consecutive days to induce gingival inflammation. After 2 weeks, 0.1 ml of a suspension of 10^9^ CFU/ml of live *P. gingivalis* (ATCC BAA-308, strain W83, a highly virulent strain [16]) suspended in PBS with 2% carboxymethyl cellulose (Sigma-Aldrich Corp, Lyon, France) was inoculated per os per animal for 5 consecutive days. Infection was repeated 3 times a week until the end of the experimental period (20 weeks) which started at the first *P. gingivalis* inoculation. After 12 weeks, 15 controls and 15 periodontitis-affected mice were killed to evaluate their macroscopic alveolar bone status. The remaining periodontitis-affected mice were then separated into 2 groups. One group of 15 mice received weekly intramuscular injections of saline for 8 weeks (sham-treated periodontitis group, referred below to as the perio group). The other group of 30 mice received weekly intramuscular injections of RGTA OTR4120 in saline (1.5 mg/kg, a gift of OTR3, Paris, France) for 8 weeks (RGTA-treated group, referred below at as the RG group). Control (n = 15), perio (n = 15) and RGTA-treated (n = 30) mice were killed after 20 weeks (12 weeks of disease induction followed by 8 weeks of treatment). The whole experimental protocol is shown in Fig. 1.

Of note, RGTA are polymers that carry carboxymethyl and sulfate groups on a dextran backbone. They have unique properties including resistance to degradation, binding and protection of ECM structural and signaling proteins, and specific localization to sites of inflammation [9, 17]. Their synthesis and structure have been described elsewhere [18].

In humans, RGTA are administered locally for the treatment of cornea and skin [9, 10]. However, due to the small size of the mouse a topical local application was not possible; in addition, intramuscular injections were the only way to be certain of the exact dose to administer.

### 2.2 Sample processing

At the end of their respective experimental periods, the mice were killed by cardiac exsanguination.

### 2.3 Detection of serum *P. gingivalis* antibodies

At the end of the 20-week period, blood samples were collected from the 3 groups by intracardiac puncture. Sera were stored at −80 °C until use. Immunoglobulin G antibodies specific to lipopolysaccharide (LPS) of *P. gingivalis* were measured using a homemade ELISA. The wells of 96-well flat-bottom microtiter plates were coated in triplicates with *P. gingivalis* LPS. After washing and blocking the plates, serum samples were added to individual wells and specific mouse IgG antibodies were detected with an alkaline phosphatase-conjugated antimouse immunoglobulin. The absorbance was read at 405 nm using an ELISA plate reader [19].

### 2.4 Alveolar bone loss measurements

**Fig. 2:**
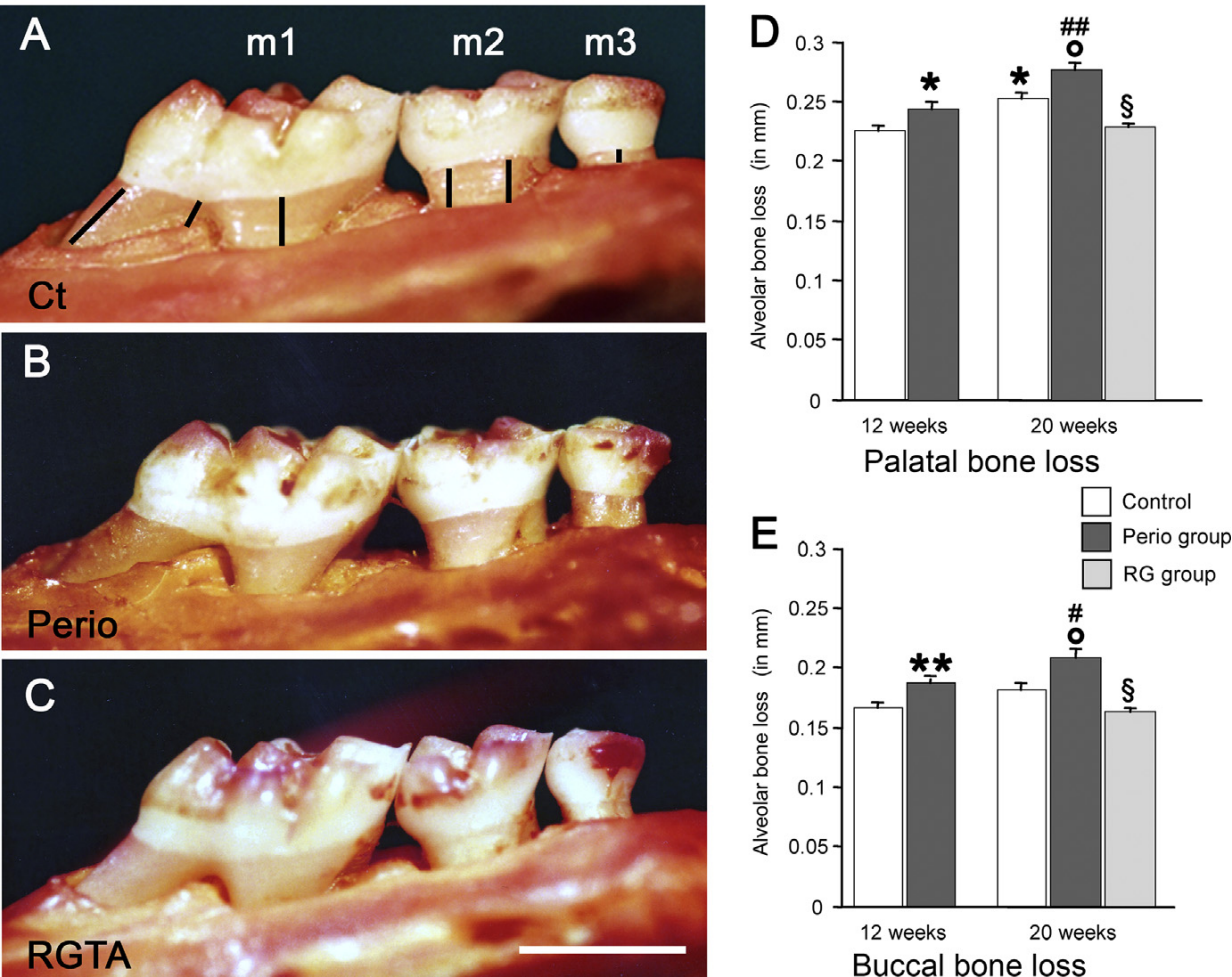
Evaluation of the macroscopic bone loss in the right maxilla molars. Bone loss at the palatal aspect in control (Ct, A), Perio (B) and RGTA-treated (C) groups. Bone loss measurements were taken on the three molars as shown in A from the cemento-enamel junction to the alveolar bone crest. Bone loss was particularly increased on m2 (second molar) and m3 (third molar) in the perio group. Note the bone restoration in the RGTA-treated group. Bar on C = 1 mm. D. Quantitative palatal bone loss. E. Quantitative buccal bone loss in Control, Perio, and RG group at 12 and 20 weeks. * *P* < 0.005, ** *P* < 0.0001 versus the corresponding control group. ° *P* < 0.03 vs the 20-weeks control group. # *P* < 0.05, ## *P* < 0.005 vs the 12-weeks perio group. § *P* < 0.0001 vs the 20 weeks perio group.

The right maxillas of the 12 and 20-week samples were de-fleshed after a 10-minute treatment in boiling water, immersed overnight in 3% hydrogen peroxide, and stained in red using the von Gieson technique. The lingual and buccal aspects of the molars were oriented under a Tessovar Photomacrographic Zoom System (Zeiss, Oberkochen, Germany) and photographed. Six measurements per aspect were taken over the 3 molars between the cementum-enamel junction and the alveolar crest to assess macroscopic bone loss (Fig. 2A). The results are expressed as the mean of the 6 measurements.

### 2.5 Histology

Mandibles were sampled and fixed in either cold (4 °C) 70% ethanol (right hemi-mandibles) or 4% paraformaldehyde pH 7.2–7.4 (left hemi-mandibles). After processing, the bones were embedded without demineralization at −20 °C in methyl methacrylate (Merck, Darmstadt, Germany). The blocks were coded and processed for sectioning in a Polycut E microtome (Leica, Wetzlar, Germany). Series of 4 μm-thick sections were cut perpendicularly to the molar root axis.

Toluidine blue (pH 3.8) was used for general morphological examination and evaluation of gingival inflammation. Alkaline phosphatase (ALP) was revealed in osteogenic cells (preosteoblasts and osteoblasts) by incubating the sections with naphthol ASTR phosphate (Sigma-Aldrich Corp, Lyon, France) and diazoted fast blue RR (Sigma-Aldrich Corp, Lyon, France) for 30 min at 37 °C (pH 9) in the presence of MgCl_2_ (Sigma-Aldrich Corp, Lyon, France). Tartrate-resistant acid phosphatase (TRAP), a marker of preosteoclasts and osteoclasts in the bone environment, was detected using hexazotised pararosanilin and naphthol ASTR phosphate (Sigma-Aldrich Corp, Lyon, France). Nonosteoclastic acid phosphatase activity was inhibited by 50 mM L(+)-tartaric acid (Sigma-Aldrich Corp, Lyon, France). Sections were lightly counterstained with toluidine blue (pH 3.8).

A rabbit polyclonal antibody against IL-1ß (1:25, NBP1-19775, Novus Biologicals, Lille, France) was used. Ten percent normal goat serum (Eurobio, Les Ulis, France) was used to reduce nonspecific background staining. The sections were incubated with the primary antibody for 20 h, followed by the secondary biotinylated antibody (goat antirabbit IgG, 1:200, Vector, Burlingame, CA) for 90 min. They were then treated with 3% hydrogen peroxide (10 min) and avidin-biotin peroxidase complex (ABC Vectastain kit, Vector, Burlingame, CA) (60 min). PBS 0.1 M was used for washing steps between incubations. Diaminobenzidine tetrahydrochloride (Sigma-Aldrich Corp, Lyon, France) was used as a chromogen. The sections were counterstained with light toluidine blue (pH 3.8). Negative controls were prepared by omitting the primary antibody, by replacing the primary antibody with nonimmune serum at the same dilution, or by using an irrelevant secondary antibody.

### 2.6 Histomorphometry

Morphometry was performed at a constant magnification (260x) and analyzed with Image J software (NIH, Bethesda, MD, USA). The following parameters were measured in the zone immediately above the alveolar bone: 1/the total pocket area [pocket epithelium + subepithelial infiltrated connective tissue (ICT) around the first molar; expressed in mm^2^]; 2/the ICT area (in mm^2^). This enabled us to calculate the pocket epithelium area (in mm^2^); 3/the number of IL-1ß + cells in the pocket tissues of the interdental septum between the first and second molars (N/mm^2^).

Bone reactions were evaluated along the alveolar wall of the third molar where the following parameters were quantified: 1/the length of the socket wall, 2/the resorption surface (Oc.S/BS), expressed as percentage of the socket wall; 3/the number of on-bone osteoclasts (cells in contact with the bone surface) per mm of bone surface (N.Oc-on/BPm); 4/the number of off-bone osteoclasts (cells located some distance from the bone surface) per mm of bone surface (N.Oc-off/BPm). This enabled us to calculate the total number of osteoclasts along the socket wall (N.tot Oc/BPm) and the on-bone/off-bone osteoclasts ratio; 5/the mean number of nuclei per on-bone osteoclast, which is an accurate index of cell activity. Data and terminology were standardized according to the recommendations of the American Society for Bone and Mineral Research nomenclature committee [20].

### 2.7 Statistics

Data were compared using nonparametric tests (Kruskal Wallis test followed, if significant, by group wise comparisons with the Mann-Whitney U test). Differences were considered significant when p < 0.05. Data are given as mean ± SEM.

## 3 Results

### 3.1 Serum antibodies against *P. gingivalis*

The two *P. gingivalis*-infected groups had elevated serum antibodies against the bacteria (control group: 1.3 ± 0.2; perio group: 29.0 ± 2.9; RG group: 33.2 ± 0.7). The differences were significant (perio group: p < 0.005; RG group: p < 0.0001 vs the control group, respectively). The perio and RG groups were not significantly different.

### 3.2 RGTA treatment reduces alveolar bone loss to control levels

Alveolar bone loss (Fig. 2A–C) was evaluated on the buccal and palatal aspects of the right maxilla. Bone loss increased in the controls between the 12th and 20th weeks and was significant only on the palatal aspect (+11%, p < 0.005. Fig. 2D).

At 12 and 20 weeks bone loss was higher in the perio groups than in the respective corresponding controls on the buccal aspect (+12% at 12 weeks, p < 0.005; +15% at 20 weeks, p < 0.03, respectively. Fig. 2E) and on the palatal aspect (+7% at 12 weeks, p < 0.02 and +14% at 20 weeks, p < 0.03, respectively. Fig. 2D). Strikingly, 8 weeks of RGTA treatment induced a gain of bone height of 28% on the buccal aspect (p < 0.0001) and of 21% (p < 0.0001) on the palatal aspect compared with the corresponding perio groups (Fig. 2D & E). Notably, alveolar bone loss returned to the level measured in the 12 weeks controls (Fig. 2D & E).

### 3.3 Gingival inflammation decreases after RGTA treatment

**Fig. 3:**
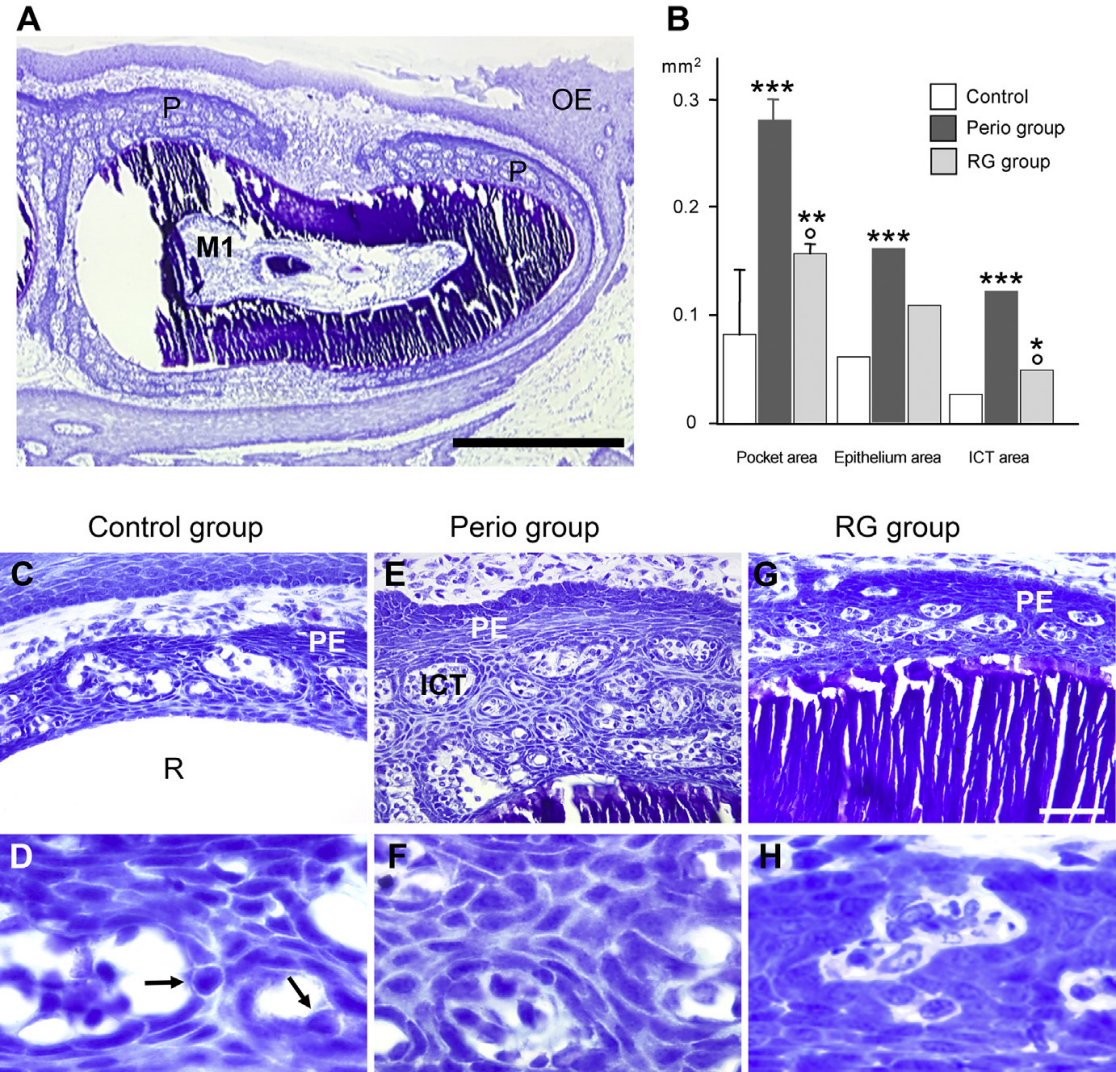
Changes in gingival inflammation. A. General view of the pocket tissues around the mandible first molar (M1) in a sham-treated mouse. P: pocket tissues (pocket epithelium + infiltrated connective tissue, ICT); OE: oral epithelium. Bar = 0.5 mm. B. Quantitative changes in pocket tissues at m1. In a control sample the pocket tissues (C) are rather thin over the root (R). At higher magnification, the intercellular spaces of the pocket epithelium (PE) are enlarged. Inflammatory cells (arrows) are crossing it (D). In a perio animal, the pocket tissues are thick and contain many large ICT islets (E). The intercellular spaces of the pocket epithelium are uniformly wide (F). The RGTA treatment reduces the pocket tissues and the size of the ICT islets (G). The intercellular spaces are tight (H). Same magnification from C to E (bar = 50 μm) and from F to H (bar = 20 μm). * *P* < 0.05, ** *P* < 0.003, ***P < 0.002 vs the control group; ° *P* < 0.01 vs the perio group.

Thin and superficial pocket tissues (pocket epithelium + subepithelial ICT) were present in the controls on the three molars. In the perio group, thick pocket tissues surrounded the three molars (Fig. 3A) and extended more apically than in the controls. In the RG group, pocket tissues were thinner and extended less profoundly than in the perio group.

The mean pocket tissue (epithelium + ICT) area increased by 340% in the perio group (p < 0.002 vs the control group). RGTA treatment decreased this parameter by 44% compared with the perio group (p < 0.01); however, it remained 190% higher than the score of the control group (p < 0.03).

The mean pocket epithelium area was 280% larger in the perio group than in the control group (p < 0.002); it did not significantly decrease in the RG group. The mean ICT area strongly increased by 480% in the perio group versus the control group (p < 0.002). RGTA treatment reduced ICT area by 60% versus the perio group (p < 0.01). However, it remained higher than in the control group (+190%; p < 0.05) (Fig. 3B).

The intercellular spaces of the pocket epithelium were wide and frequently contained migrating inflammatory cells, mainly polymorphonuclear leukocytes, in the control (Fig. 3C & D) and perio (Fig. 3E & F) groups. In contrast, intercellular spaces were tighter, with less infiltrating cells in the RG group (Fig. 3G–H).

### 3.4 Marked decrease of IL-1ß+ cells in RGTA treated mice

**Fig. 4:**
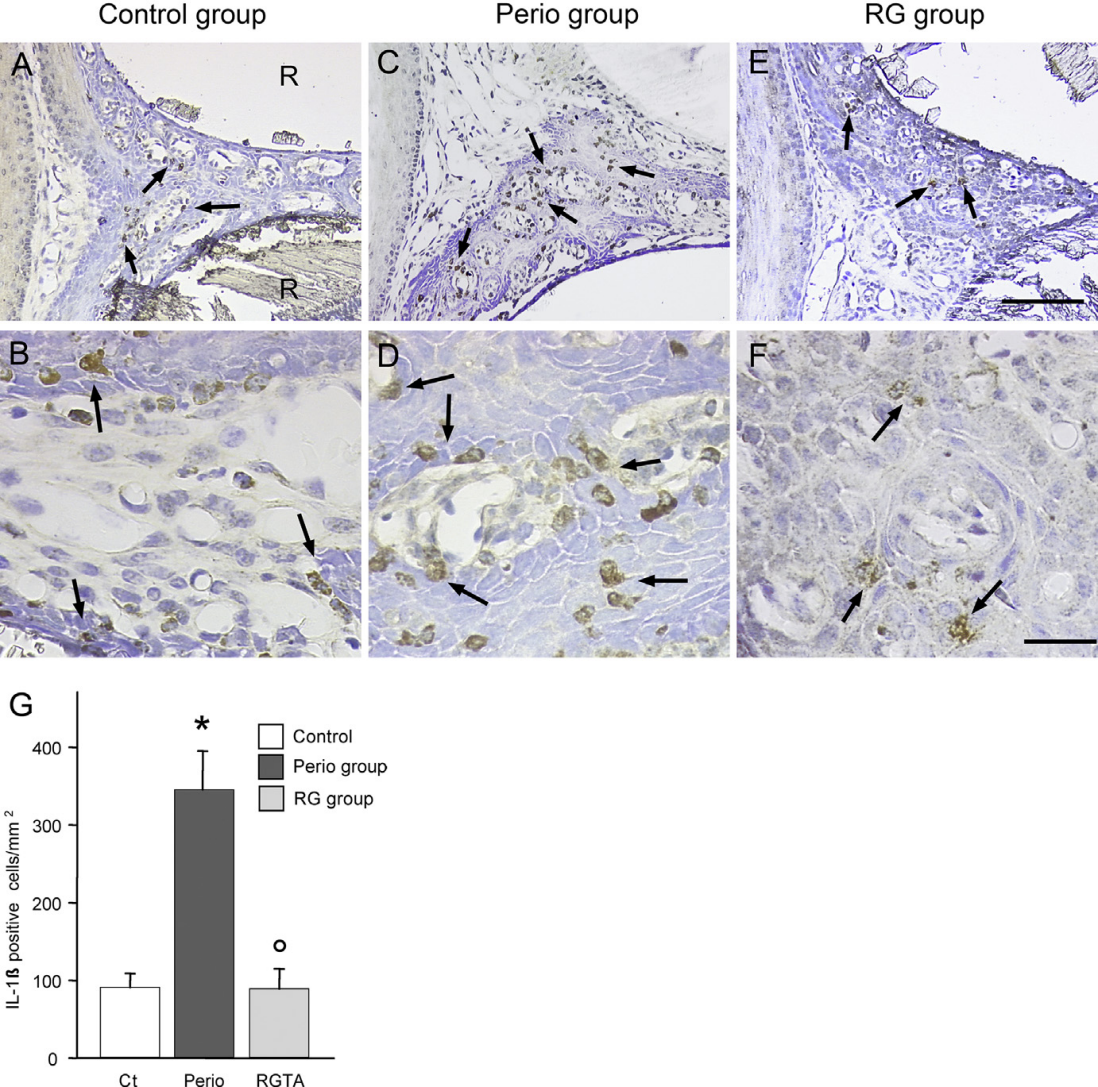
Variations in IL-1ß positive cells. Interdental zone between the first and second molars. A. General view in a mouse of the control group. B. Control group, higher magnification. C. Perio group, general view. D. Perio group, higher magnification. E. RG group, general view. F. RG group, higher magnification. IL-1ß positive cells (arrows) are scattered in the ICT and the pocket epithelium. R: dental root. Same magnification in A, C and D; bar = 200 μm. Same magnification in B, D and F; bar = 50 μm. G. Quantitative evaluation. * *P* < 0.002 vs the control group; ° *P* < 0.002 vs the perio group.

Control tissues showed few IL-1ß positive cells (Fig. 4A & B). As expected, their numbers strongly increased in the perio group (Fig. 4C & D). Remarkably, the RG group showed a profile similar to the control group (Fig. 4E & F). The IL-1ß-positive cells were located in the ICT and in the pocket epithelium (Fig. 4B & F). Some cells were inside or migrating through the ICT vessels. No attempt was made to identify the IL-1ß positive cells.

The number of IL-1ß positive cells increased by 392% in the perio group versus the control group (p < 0.002). In contrast, it decreased by 75% in the RG group compared with the perio group (p < 0.002) (Fig. 4G).

### 3.5 Osteoclastic bone resorption significantly decreases after RGTA treatment

**Fig. 5:**
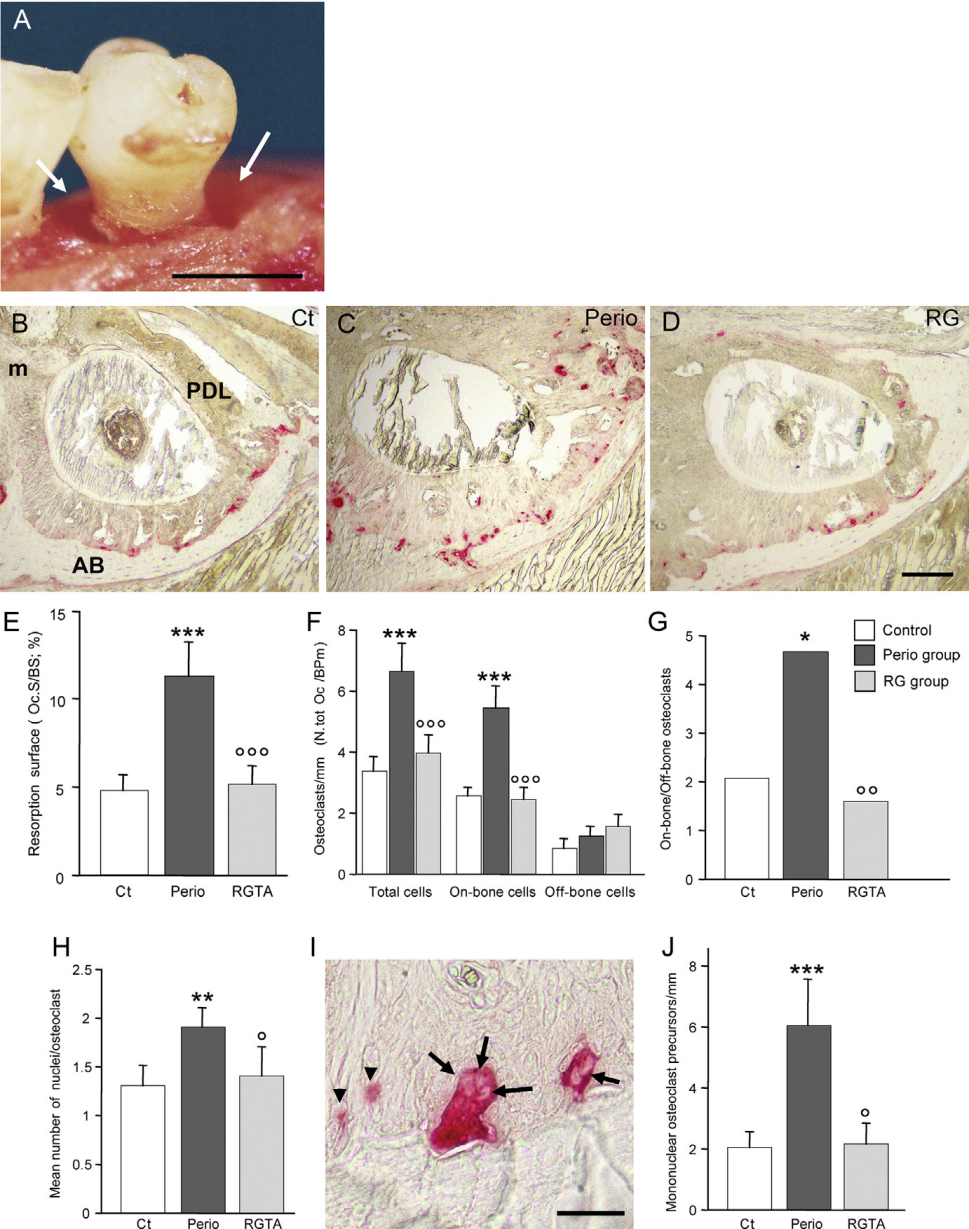
Evaluation of osteoclastic bone resorption. A. Mice of the perio group. Macroscopic view of a third molar where a circumferential intrabony lesion develops (arrows). B to D. Alveolar wall of the socket of the third molar. TRAP enzymostaining reveals the cells of the osteoclastic lineage in red. The cells present in the control (Ct) sample are engaged in the physiological drift of the tooth (B). The ongoing periodontitis destruction (C) superimposes on this physiological cell pool. Restoration of the physiological resorption with the RGTA treatment (D). m: mesial aspect of the socket. E. Changes in resorption surface (Oc.S/BS).F. Quantitative changes in osteoclasts. Active (on-bone) and inactive (off-bone) osteoclasts were counted separately. This allowed us to calculate the total number of osteoclasts (N.tot Oc/BPm) recruited in the site. G. Changes in the on-bone/off-bone ratio, an index of osteoclast activation. H. Changes in mean number of nuclei per osteoclast, an index of cell activity. I. Nuclei in resorbing osteoclasts (arrows) in an animal of the perio group. The arrowheads point out to mononucleated preosteoclasts. J. Variation in mononucleated preosteoclasts. Bar in A: 0.5 mm. Same magnification from C to E; bar = 100 μm. Bar in I = 20 μm. * *P* < 0.03, **P< 0.02, ***P < 0.005 vs the control group; ° *P* < 0.05, °° *P* < 0.03, °°° *P* < 0.02 vs the perio group.

Osteoclastic bone resorption was evaluated along the socket of the third molar where a circumferential vertical bone defect typically developed in the perio group at the expense of the socket wall, enlarging the periodontal ligament space (Fig. 5A). Along the tooth socket, resident osteoclasts allow for the physiologic drift of the tooth (Fig. 5B); during periodontitis, inflammation induces the recruitment of additional osteoclasts, which combined with the action of the resident osteoclast population, lead to pathological bone loss. Frequently, the mesial aspect of the socket was lost (Fig. 5C). In the perio group the osteoclasts appeared to be larger than their control counterparts. RGTA treatment re-established a resorption profile similar to that of the controls (Fig. 5D).

In the perio group, the bone resorption surface (Oc.S/BS) was increased by 2.5-fold compared with the control group (p < 0.005; Fig. 5E). Accordingly, the number of total osteoclasts (N.tot Oc/BPm) present along the socket was 200% higher in the perio group than in the control group (p < 0.005; Fig. 5F). When the osteoclasts were separated into resorbing (on-bone) and non-resorbing (off-bone) cells, the on-bone cells were strongly increased (+217 %, p < 0.005 vs the controls) while the off-bone cells were not modified (Fig. 5F). Consequently, the on-bone/off-bone ratio was increased by 230% in the perio group (Fig. 5G). The resorption potential of the resorbing osteoclasts was evaluated by calculating the mean number of nuclei per cell. It was increased by 143% in the perio group versus the control group (p < 0.02) (Fig. 5H & I). Remarkably, RGTA treatment restored the resorption surface to the control level (- 53%, p < 0.02 vs the perio group; Fig. 5E), resulting in a marked decrease versus the perio group in total (- 42%, p < 0.02) and on-bone osteoclasts (- 56%, p < 0.02) (Fig. 5F) and restoration of the on-bone/off bone ratio (Fig. 5 G). The mean number of nuclei per osteoclast returned to the control value (- 26%, p < 0.05 vs the perio group) (Fig. 5H). Moreover, the mononucleated preosteoclasts (Fig. 5I & J) were increased in the perio group (+300%, p < 0.005 vs the control group) and reduced in the RG group (- 66%, p < 0.05 vs the perio group).

### 3.6 RGTA treatment promotes bone formation

**Fig. 6:**
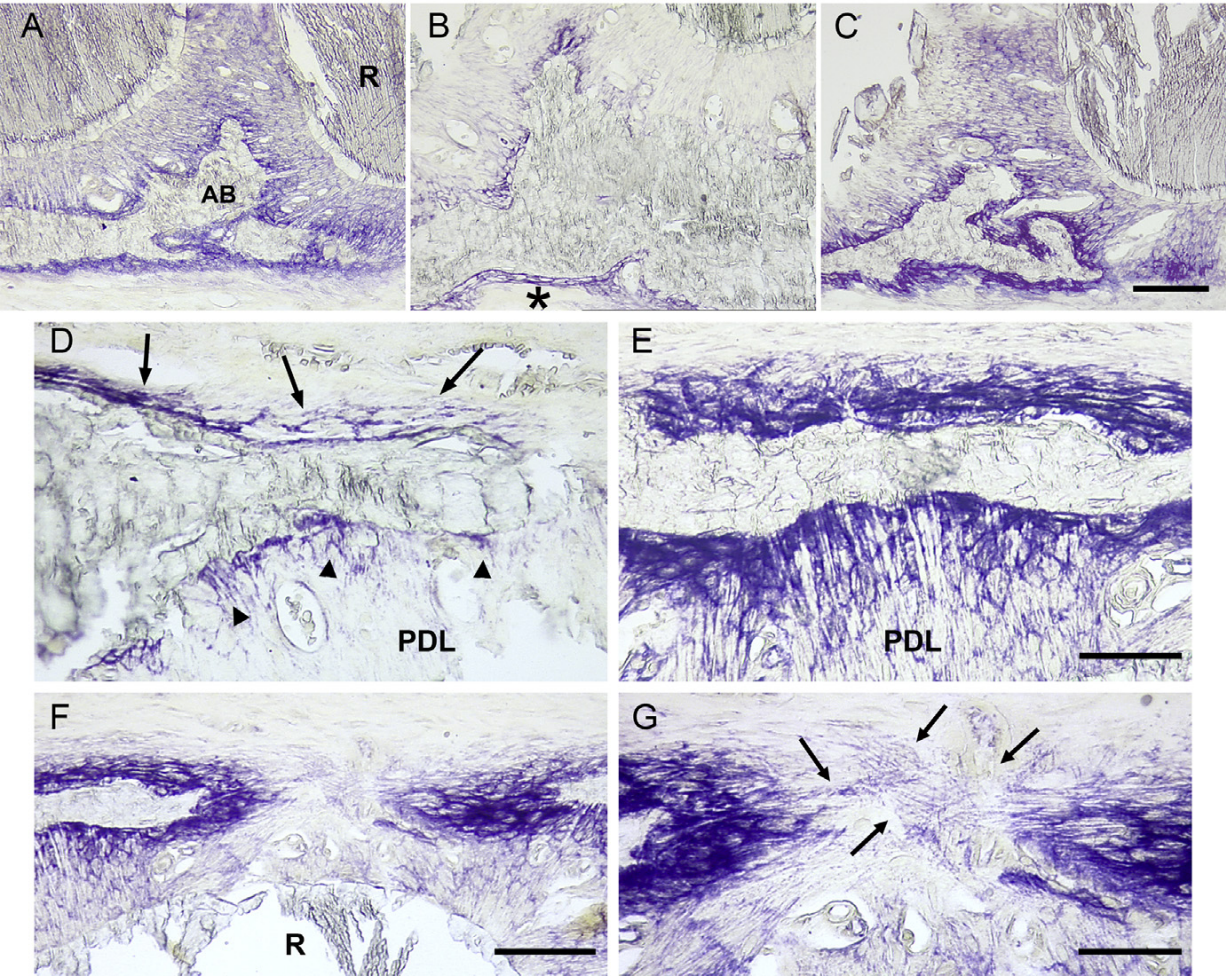
Bone formation. Bone formation was evaluated after enzymochemistry for alkaline phosphatase (ALP) which stains osteogenic cells in purple. A to C. Osteogenic cells at the interdental septum between the first and third molars. A. Control animal. The bone surfaces are covered by a continuous layer of positive cells. Bo: bone; R: root. B. Perio group. The staining has almost completely vanished along the alveolar walls. A thin positive layer subsists along the cortical suface (asterisk). C. The RGTA treatment restored a thick layer of positive cells along all the bone surfaces. D. Perio sample, the layers of ALP-positive cells are thin and discontinuous (arrows); the staining of Sharpey's fibers is weak (arrowheads). PDL: periodontal ligament. E. RGTA-treated sample, the layers of positive cells are thick and the staining is strong, indicating intense recruitment of osteogenic cells. Note the strong staining of the Sharpey's fibers of the periodontal ligament. F. RGTA treatment. Strong recruitment of osteogenic cells to fill a cortical defect. R: root. G. Higher magnification of F. ALP-positive fibers bridge the defect (arrows), they may guide the cells filling the defect. Same magnification from A to C (bar = 200 μm) and in D and E (bar = 100 μm). Bar in F = 200 μm, in G = 100 μm.

Bone formation was evaluated using ALP enzymochemistry to reveal the osteogenic cells (osteoblasts and osteoprogenitors). In the control animals, several layers of ALP + cells lined the periosteum, the vascular channels, and the socket (Fig. 6A). The Sharpey's fibers were positive for ALP from their emergence of the socket wall. In the perio group, many bone surfaces lacked ALP-positive cells. When present, the ALP-positive cell layer was thin and weakly stained (Fig. 6B). In contrast, thick layers of strongly stained ALP-positive cells lined the bone surfaces in the RG group (Fig. 6C).

ALP also coats the Sharpey's fibers in the periodontal ligament [21] (Fig. 6A). The Sharpey's fibers were not coated with ALP (Fig. 6D). Instead, they were strongly stained as well (Fig. 6E).

Where the disease had interrupted the continuity of the cortex, thick layers of osteogenic cells filled the defect (Fig. 6F). When the gap between the edges of the defect was not yet closed, thin ALP-positive fibers joined the edges, as if preparing the path for the migration of osteogenic cells (Fig. 6G).

## 4 Discussion

In a model of *P. gingivalis*-infected mice, RGTA treatment was able to control gingival inflammation, to stop inflammation-driven bone resorption and to boost bone formation leading to alveolar bone height and contour restoration. Notably, this occurred while *P. gingivalis* infection was maintained by repeated inoculations until the end of the experiment. This demonstrates that breaking the vicious cycle of chronic inflammation is a realistic goal to treat periodontitis destruction and to regenerate the periodontium, eliminating the need for surgical intervention.

In our experience, the protocol presented by Baker et al. [22] to implant *P. gingivalis* in the mouth of Balbc/cByJ mice failed to provoke bone loss, as previously reported [23]. Thus, to obtain bone loss, we first orally implanted a strain of *A. viscosus* in an attempt to mimic the human condition in which *P. gingivalis* colonizes the gingival crevice after inflammation-promoting bacteria initiate gingival injury [24, 25]. In a pilot study we observed that *P. gingivalis* infection had to be sustained until the end of the experimental period, since interrupting the infection did not allow for increased bone loss. The presence of high levels of serum antibodies against *P. gingivalis* in the perio and RG-treated groups shows that our modified protocol was effective in implanting the bacteria.

In addition to evaluating the standard histological gingival signs of inflammation such as pocket epithelium proliferation, increase in ICT and invasion of inflammatory cells, IL-1ß was selected as a marker of inflammation, as this cytokine is predominantly expressed in the gingival tissues during the onset and progress of periodontitis [15], particularly in active sites [26, 27]. Moreover, *P. gingivalis* promotes IL-1ß gene expression and production [28, 29].

While control animals showed signs of gingival inflammation, *P. gingivalis* implantation worsened the gingival condition, as shown by epithelium pocket proliferation and edema, increase in ICT and intense infiltration of IL-1ß-positive cells. RGTA treatment reversed gingival deterioration as it strongly reduced the ICT and improved the status of the pocket epithelium in which the intercellular spaces were tighter than in the control and perio groups, showing that it was less permeable. The decrease in infiltrating IL-1ß positive cells confirmed the control of local inflammation and possibly of the systemic reaction to *P. gingivalis* implantation. These data are in agreement with previous observations in another model of periodontitis [12, 13]. The effect of RGTA treatment was similar to the in vitro HS treatment of endotoxin-treated chondrocytes, which reduced expression of several cytokines including IL-1ß and proteolytic enzymes [30].

During periodontitis, bone destruction results from an imbalance between bone formation that is inhibited and bone resorption that is increased [31, 32]. *P. gingivalis* infection had a strong impact on bone metabolism.

*P. gingivalis* factors promote bone resorption [28, 29, 30, 31, 32, 33, 34, 35, 36]. In this study, bone resorption was evaluated along the socket of the third molar; the resorption provoked by *P. gingivalis* infection added to the physiological osteoclastic resorption related to the physiological tooth drift [37]. The number of osteoclasts doubled in the infected untreated animals, resulting in a strong increase in resorption surface. Recruited osteoclasts were actively engaged in resorption, as demonstrated by the strong increase in the on-bone/off bone osteoclast ratio. In addition, the resorption potential of each osteoclast was augmented, as shown by the increase in mean number of nuclei per active cell, a parameter that reflects individual cell activity [38, 39]. The resorption potential was also high since the number of mononucleated preosteoclasts, either about to fuse, to form new osteoclasts, or to be incorporated in existing osteoclasts, was strongly increased. In fact, all the resorption parameters converged to generate powerful alveolar bone destruction. Strikingly, RGTA treatment reversed all the osteoclastic resorption parameters that regained their basal values, indicating that the treatment had not only stopped the destructive phase, but also allowed the regeneration process to begin.

The thickness of the osteogenic cell layer (osteoblasts and osteoprogenitors) was reduced in the perio group, demonstrating a reduction in the number of available cells, which in turn jeopardized the rebuilding of alveolar bone. The expression of the osteogenic marker ALP was weak, indicating that the intrinsic activity of the remaining osteogenic cells was also affected. Accordingly, in vitro *P. gingivalis* endotoxin represses the osteoblastic phenotype by inhibiting the expression of phenotypic markers, including ALP, and the formation of bone nodules [40]. In addition, the ALP coating of the Sharpey's fibers was lost, indicating that the metabolic integrity of the periodontal ligament was affected and shifted from an anabolic state to a catabolic-oriented condition. RGTA treatment thickened the osteogenic layers and strongly enhanced ALP expression, in agreement with our previous observation [12]. The enhancement of osteogenic cell recruitment and the intensity of their phenotypic expression (confirmed by their ALP expression) provided the basis for a potent formation reaction. Accordingly, this resulted in the regeneration of the alveolar bone in height and shape. In fact, the macroscopic bone gain reset the anatomy of the alveolar bone back to that of the 12-week control mice, without bone over-formation or anatomical deformity. The thickness of the osteogenic layer at the time of sacrifice indicated that the dynamics of bone formation was still strong. Moreover, a thick ALP coating embedded the Sharpey's fibers, showing the re-orientation of the PDL to an anabolic state.

These results confirm our previous observations in another model of periodontitis [12, 13]. More generally these outcomes are in line with our data showing that RGTA are regulators of bone resorption and strong promoters of bone formation by recruiting osteoblast-committed precursors from their anatomical niches [13, 41, 42]. The effects of RGTA treatment on bone reaction are consistent with the known properties of HS. On one hand, HS control bone resorption by inhibiting RANKL-induced osteoclast differentiation and activity [43], adhesion of osteoclast precursors and spreading of differentiated osteoclasts [44]. On the other hand, HS stimulate mesenchymal stem cell proliferation, their commitment to the osteoblastic phenotype and their ability to form bone when implanted in bone defects [45, 46].

RGTA are carboxymethyl sulfate polymers prepared from dextran that are resistant to mammalian enzyme [47]. Like HS, they form complexes with heparin-binding molecules. They provide a scaffold and replace the destroyed HS by binding to available heparin-binding sites of structure molecules, such as fibrous collagens, fibronectin and laminin, which they protect from further degradation [9, 10]. This provisional ECM architecture restores a proper microenvironment providing a spatial organization in which heparin-binding communication factors can be re-positioned and protected at the right location and are available when needed. In vitro, RGTA protect, stabilize and induce slow release of a- and bFGF [48]. During the healing of diabetic ulcers, RGTA treatment increases the skin content of VEGF and TGF-ß1 without affecting gene transcription, thus highlighting their sequestering, protecting and stabilizing effects on the growth factors present in the wounds [49]. This microenvironment mimics pre-injury conditions, allowing for the control of inflammation, including the reduction in inflammatory cell infiltration [50], and the restoration of a microcellular environment providing adequate cell-cell and cell-ECM communication. This allows the reformation of a tissue-specific ECM and the original architecture of the injured organ [51]. RGTA efficacy on inflammation resolution and on wound healing has been tested in several models including skin and bone lesions [41, 42, 49, 50, 51, 52], mucositis [48, 53], and periodontitis [12, 13, 14].

## 5 Conclusion

In conclusion, these results confirm that RGTA are able to control the chronic inflammation characteristic of periodontitis and guide periodontal regeneration by allowing the tissue's intrinsic potential for repair to take place, which is inhibited by the disease. They also demonstrate that despite the absence of bacteria control, the tissues retain their potential to spontaneously recover their anatomical and metabolic characteristics. These preclinical studies suggest that RGTA may be a good candidate to treat human periodontitis, thus avoiding surgical intervention and support the development of RGTA for clinical use in humans.

## Declarations

### Author contribution statement

Benjamin R. Coyac, Laurent Detzen, Philippe Doucet, Brigitte Baroukh, Annie Llorens: Performed the experiments.

Martine Bonnaure-Mallet: Contributed reagents, materials, analysis tools or data.

Marjolaine Gosset: Conceived and designed the experiments.

Denis Barritault, Marie-Laure Colombier: Conceived and designed the experiments; Analyzed and interpreted the data.

Jean-Louis Saffar: Conceived and designed the experiments; Analyzed and interpreted the data; Wrote the paper.

### Funding statement

This work was supported by a Grant from the Agence Nationale de la Recherche (ANR-07-RIB-022-02), France.

### Competing interest statement

The authors declare the following conflict of interests: Denis Barritault is the co-inventor and co-owner of the patents describing the RGTA technology and president of the company OTR3, manufacturer of RGTA-based products. The other authors declare no conflict of interest.

### Additional information

No additional information is available for this paper.
